# Mobile Health Applications to Promote Active and Healthy Ageing

**DOI:** 10.3390/s17030622

**Published:** 2017-03-18

**Authors:** Jorunn L. Helbostad, Beatrix Vereijken, Clemens Becker, Chris Todd, Kristin Taraldsen, Mirjam Pijnappels, Kamiar Aminian, Sabato Mellone

**Affiliations:** 1 Department of Neuroscience, Faculty of Medicine, Norwegian University of Science and Technology, 7491 Trondheim, Norway; beatrix.vereijken@ntnu.no (B.V.); kristin.taraldsen@ntnu.no (K.T.); 2 Robert Bosch Foundation for Medical Research, 70184 Stuttgart, Germany; Clemens.Becker@rbk.de; 3 School of Health Sciences, University of Manchester, and South Manchester University Hospital NHS Trust, and Manchester Academic Health Sciences Centre, Manchester M13 9PL, UK; chris.todd@manchester.ac.uk; 4 Department of Human Movement Sciences, Vrije Universiteit Amsterdam, Amsterdam Movement Sciences, 1081BT Amsterdam, The Netherlands; m.pijnappels@vu.nl; 5 Laboratory of Movement Analysis and Measurement, Ecole Polytechnique Federale de Lausanne, 1015 Lausanne, Switzerland; kamiar.aminian@epfl.ch; 6 Department of Electrical, Electronic and Information Engineering, University of Bologna, 40126 Bologna, Italy; sabato.mellone@unibo.it

**Keywords:** healthy ageing, mobile technology, mobile health applications, activity monitoring, smartphones, smartwatches, wristbands

## Abstract

The European population is ageing, and there is a need for health solutions that keep older adults independent longer. With increasing access to mobile technology, such as smartphones and smartwatches, the development and use of mobile health applications is rapidly growing. To meet the societal challenge of changing demography, mobile health solutions are warranted that support older adults to stay healthy and active and that can prevent or delay functional decline. This paper reviews the literature on mobile technology, in particular wearable technology, such as smartphones, smartwatches, and wristbands, presenting new ideas on how this technology can be used to encourage an active lifestyle, and discusses the way forward in order further to advance development and practice in the field of mobile technology for active, healthy ageing.

## 1. Demographic Changes and the Need for Development of New Health Services

The European population is rapidly ageing [1]. Average life expectancy has exceeded 80 years, which is an increase of ten years since 1970. Those aged 80 and over are the fastest growing, and are expected to represent 20% of the older population by 2050 [2]. Many of the additional years will be lived with chronic conditions [3]. Increasing life expectancy together with decreasing fertility rates will reduce the number of economically active persons per pensioner in the years to come, thus changing the old age dependency ratio and thereby putting more pressure on the health care system. This necessitates a shift from treatment towards prevention of age-related diseases and a need to develop innovative solutions that enable the ageing generation to stay independent longer and empower them to take care of their own health and function. In this paper we focus on the possibilities that new health technology, based on wearable technology, such as smartphones, smartwatches, and wristbands, can offer, the content and functionality of such applications, ideas on how such technology can be used to encourage an active lifestyle, and the way forward with regard to further development of such technologies and services.

## 2. Promoting Active, Healthy Ageing

Ageing is often accompanied by an increased risk of age-related diseases and decline in function. This decline can be related to the ageing process, itself, with structural and functional changes in the brain and in cardiovascular, skeletal, and muscular systems. However, there is large diversity in functional levels at old age, and studies consequently find lifestyle to be an important determinant of function [4], underlining the need for personalised preventive interventions that are aimed at adoption of more healthy behaviour. For the coming decades, there is a societal goal to maximise the number of people who experience positive trajectories of ageing. To that end, innovative interventions tailored to individual functional levels and focusing on healthy lifestyles are warranted.

Inactivity is the fourth leading cause of death worldwide [5] and increases the risk of adverse health outcomes, such as shortened life expectancy, cardiovascular disease, diabetes, and cancer [6]. Older adults are at particular risk of inactivity. Decline in physical activity is also a consistent observation in epidemiological literature on ageing and is associated with low social function, depression, and cognitive decline [7]. In order to promote active and healthy ageing and permit people to live independent lives longer, a focus on physical, social, and cognitive activity is needed.

Promoting active and healthy ageing includes early detection of risk factors for functional decline, before performance of everyday life activities starts to deteriorate notably. Studies have pointed out that activity levels drop significantly when people retire [8]. At present, retirement in Europe is typically between 60 and 68 years. Women tend to retire earlier than men, and poor health is often a factor for early retirement. By identifying early markers for decline in function and targeting interventions at reducing the risk, functional decline can be prevented in a large portion of the ageing population at the time of retirement.

According to the World Health Organisation’s (WHO) [4] report on ageing and health, doing more of what already is being done or improving existing health care systems is not enough to promote active and healthy ageing. To this end, WHO argues, a systemic change will be needed. It is recommended to develop strategies that engage people when they still have a high and relatively stable functional capacity. This may imply development of health care services designed for use by the people themselves to be initiated long before they enter traditional health services delivered by health care personnel. Such services have the potential to empower people to be in control of their own health and function in order to stay healthy and independent as long as possible.

## 3. Mobile Health Technology and Services

Mobile health, or mHealth, is an aspect of electronic health or eHealth that focuses on the delivery of health care services via mobile communication devices [9]. There is no unified definition of mHealth, but WHO has described it as “medical and public health practice supported by mobile devices, such as mobile phones, patient monitoring devices, personal digital assistants (PDAs), and other wireless devices”. It is based on use of mobile technology or wireless devices and sensors that are intended to be worn, carried, or accessed by the person during normal daily activities [9]. Technology to be worn on the body (wearables), such as smartphones, smartwatches, and wristbands, also include a variety of sensors that support new methods for continuous monitoring of biological, behavioural, or environmental data, delivering interventions, and assessing their outcomes. Such sensor systems include accelerometers, gyroscopes, magnetometers, barometers, sensors for the measurement of heart rate and galvanic skin response, cameras, as well as geo-sensors (GPS) for tracking one’s exact geographical position. Through such sensor systems, monitoring of aspects related to health can be performed at high precision and sampling frequency and over longer time periods than more traditional methods. Such systems are also well suited for delivering digitised interventions, as well as for self-assessment of aspects of behaviour. Through the development of algorithms derived from the sensor data and additional self-reported data, precise, and also new, information about physiological, psychological, emotional, and environmental states can be derived [10]. Use of mobile technology also offers new solutions for the delivery of health services, including the use of individualised feedback based on monitoring of behaviour in ecologically-valid contexts.

The potential for development of new services based on such technology is tremendous. In 2014 over 75% of those 65 and older in the US had a mobile phone, and over 50% used smartphones or tablets [11], while in the UK in 2012 roughly 50% used internet, which is projected to rise to 90% by 2020 [12]. The number of people worldwide owning a smartphone has reached 2.1 billion in 2016, and the numbers are expected to rise to 2.5 billion by 2020 [13].

mHealth opens up the possibility of health monitoring at both an individual and population level, and can encourage healthy behaviours to prevent or reduce health problems [14]. With the increasing global access to mobile technology, there is an enormous potential for the development of mHealth applications for the private market. This is also reflected by the rapidly increasing number of applications that are developed by large actors in the market, such as Apple [15], Google [16], Phillips [17], and Samsung [18]. The numbers of health and fitness apps in Apple’s App Store and Google Play Store (Android app store) was 16,500 in 2015 [19] and the numbers are increasing rapidly.

Even though the field of mHealth applications is expanding continuously and at high speed, several limitations are still present. First and foremost, there is only modest evidence for the advantages of existing apps even though the evidence is increasing rapidly [19]. Second, most applications are one-dimensional, meaning that they address one function only, such as physical activity applications. Existing systems have not been developed based on the needs and preferences of the ageing population and thus usability, feasibility, and validity of such systems for the ageing population are not well documented. Furthermore, apps and other systems have not been strongly based on psychological theories of behaviour change and the wealth of empirical evidence that exists for how to promote uptake and maintain healthy lifestyles [20,21].

## 4. Mobile Health Technology for Disease Management and Change in Health Behaviour

mHealth applications can be divided into those aimed at disease management and those aimed at health behavioural change [22]. Several mobile phone applications and mobile phone text message interventions within disease management have been developed and tested in clinical studies, such as diabetes control [23], depression treatment [24], hypertension control [25], adherence to medication [26], and psychological support [27]. Behavioural change applications include smoking cessation by use of mobile phone text messages [28], reduction of calorie intake by use of personal digital assistant applications for diet and exercise [29], and mobile phone application interventions to increase physical activity levels [30]. A recent systematic review of the efficacy of interventions that use a variety of smart phone apps and exercise platforms to improve diet, physical activity, and sedentary behaviour concluded with modest evidence for app-based interventions [31]. It was also found that multi-component interventions were more effective than standalone app interventions. Another review from 2016 of smartphone applications aimed at promoting physical activity indicated that such apps can be efficacious in promoting physical activity, however, the effect demonstrated so far seems to be modest. An important finding was that participants at various ages and both genders responded positively to the apps, in particular those that automatically tracked activity and the progress of activity, and that were user friendly and flexible with regards to which type of activity they tracked [32].

mHealth applications aimed at increasing physical activity and facilitating behavioural change have also been developed, particularly for older people. Relatively new studies have demonstrated the effect of home-based balance and strength training (mean age 75 years) presented on tablet apps [33], and smartphone-delivered physical activity with a social element (mean age 60 years) [34]. Given the increase in the older population the next decades, mHealth interventions need to be developed with these groups of people’s needs and preferences in mind.

## 5. Development of mHealth Technologies for Older Adults

Most smartphones have not been developed specifically with older adults in mind. Even though function is highly heterogeneous at older age, there are several typical age-related changes in the cognitive (e.g., spatial orientation, memory), psychological (e.g., motivation, attitudes, beliefs), motor (e.g., movement speed, reaction time, force control, movement precision), and sensory (e.g., vision, hearing, touch sensibility) domains [35]. Studies comparing young and older adults’ use of smartphones conclude that there are five distinct human factors where older adults are different from their younger counterparts: learning time, speed of performance, error rate, retention over time, and subjective satisfaction [35]. A recent systematic literature review on older adults’ perception of technology used for fall prevention found that control, independence, and perceived need for safety are important for motivation to use the technologies [36]. Important external factors were usability, the possibility to receive feedback from the system, and costs. Another systematic literature review on technology acceptance in older adults found concerns regarding the technology, expected benefits of the technology, alternatives to technology, and social influence to be important [37]. Furthermore, the authors found that most studies had assessed technology in an early phase of the development only, and that relatively little is known still about already-implemented products.

For older adults to accept mHealth technology, it must represent a clear benefit to them and fit with their goals, expectations, and lifestyles [38]. A systematic literature review targeted at the ageing population identified independence, understanding, and visibility as facilitators for use of mHealth solutions, and complexity, limited usability, and ineffectiveness as barriers [39]. In order for use of mHealth technology in older adults to be successful in increasing uptake of interventions, the technology needs to be developed with this group’s needs and preferences in mind. To increase uptake of mHealth technology in the older generation products and services also need to be easily accessible, easy to learn to use, and to appraise critically.

An important question concerns what is required in order to develop mHealth applications that will be suitable for older adults’ needs and preferences. Co-design in technology development is an approach that actively involves all important stakeholders in order to ensure that the product meets the needs of the stakeholders and is usable [40]. This requires the use of a participatory design process with different stakeholders involved during the problem definition phase, during the development process, and in the evaluation process. For the development of mHealth technology for older adults this means that representatives for the end users, researchers, technology developers on the hardware and software side, health care providers, and companies should be involved in the entire development process. This also increases the chances for the product to reach the market [41].

Usability is one important aspect of co-design. Usability refers to the ease of use of a tool or device where the interaction between the device and the user is central [42]. Devices are developed for certain purposes and the usability refers to this specific purpose for the specific target groups using the tool or device. The International Organisation for Standardisation (ISO) defines usability as “The extent to which a product can be used by specified users to achieve specified goals with effectiveness, efficiency, and satisfaction in a specified context of use” [43]. ISO standard 9241-210, which was revised and confirmed in 2015, provides a standard for human-centred design for interactive systems. The standard describes the phases of a user-centred design process as an iterative process. Recommended phases of an iterative user-centred design process include (1) understanding and specification of the context of use; (2) specification of user requirements; (3) production of design solutions that meet user requirements; and (4) evaluation of the design against requirements.

Usability is defined by different quality indicators, such as (1) learnability, how easily users can accomplish basic tasks the first time they use the system; (2) efficiency, how fast users can perform a task after they have learned the design; (3) memorability, how easily users can re-establish good use of the system; (4) errors, referring to the number and severity of errors users make; (5) satisfaction, how pleasant users find the system; and (6) utility, the functionality of the system [42].

Different methods and approaches are used to guide and assess the usability of a system in the different phases of the user-centred design process. Common characteristics of usability tests are that participants are real users and do real tasks, that the tests include observation and recordings of what participants do and say, and that data analysis aims at revealing problems and recommends changes to fix the problems [44,45].

## 6. Development of Mobile Technology for Promoting Active and Healthy Ageing: Examples from the PreventIT Project

In order to promote active and healthy ageing, there is a need to develop health applications that focus on detection of risk related to functional decline and on interventions that promote health and prevent functional decline at older age. In what follows, we refer to the work currently being performed in the European Personalising Health and Care project, PreventIT (2016–2018, grant agreement number 689238) in order to exemplify possible development and use of mHealth technology in the context of active and healthy ageing.

**Figure 1:**
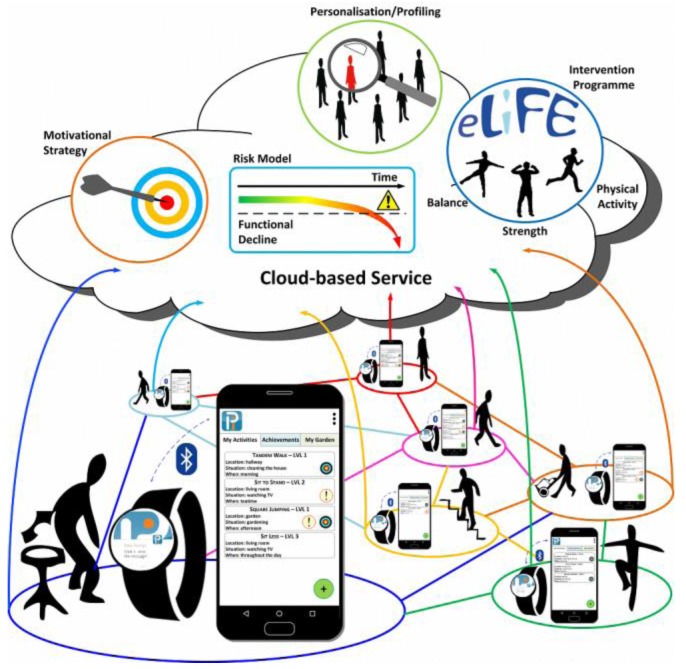
The architecture of the multifunctional PreventIT mHealth system, including risk screening for functional decline, profiling for personalisation of the intervention, an intervention with balance, strength, and physical activity integrated in daily life, and individual feedback on behaviour aimed at increasing motivation for behavioural change. A smartphone and a smartwatch are used for monitoring of behaviour, delivering the intervention, and for giving individualised feedback on behaviour.

PreventIT aims at developing and testing an ICT-based mHealth system that enables early identification of risks for age-related functional decline, and engenders behavioural change in young older adults (aged 60–70 years) in order to adopt a healthy, active lifestyle. In the project, an integrated system of a smartphone and a smartwatch will be used as the frontend technology, and a protected cloud-based solution for the handling of personal data as the backend technology. Smartphones and smartwatches are used as the basis for the development of a multifaceted tool for the assessment of risk of functional decline, for self-assessment of functional fitness, and for delivery of an individually-tailored intervention with exercises that are integrated in daily life. Active and healthy ageing requires the adoption of a healthy lifestyle over time, and often requires a lasting behavioural change. Therefore, we will also focus on how smartphones and smartwatches can be used as a platform to increase uptake of interventions and sustain motivation over time. Figure 1 shows the architecture of the mHealth system developed through the PreventIT project, including a smartphone and a smartwatch connected through a Bluetooth connection, and the processing and storing of data both in the smartphone and watch, as well as in the cloud. As the smartphone will not always be worn, behavioural information is also recorded and processed on the smartwatches and then transferred to the smartphone. In the system, results of calculated behaviour variables will be used for giving feedback on behaviour and motivational messages to the users of the system. The system is also designed to allow for social interaction between participants and offers possibilities for team competition and comparison of one’s own results with those of others.

Data are stored and processed locally on the smartphones and smartwatches, but most calculations are done using a cloud-based service. Social interaction during the intervention between participants is allowed using the cloud-based server as a communication unit.

### 6.1. Self-Assessment of Physical Performance

**Figure 2:**
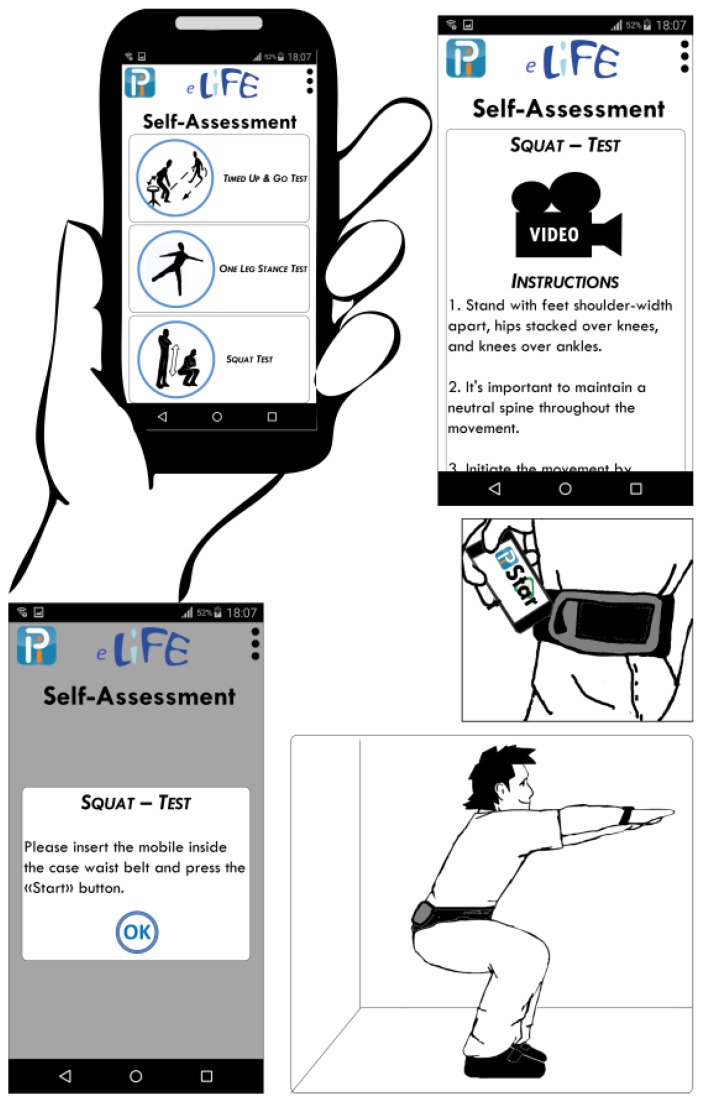
Example of a self-assessment test application for physical performance performed while having a smartphone worn in a belt around the waist. From a smartphone application different tests of physical function can be chosen, demonstrated through a video clip, and instructed by use of a virtual instructor.

Use of wearable technology offers new solutions for self-assessment of physical performance and behaviour, which, so far, to a large extent, has been assessed by professionals using a variety of clinical tests. Several test applications for use by health personnel, with wearable sensors or smartphones fixed to the body (e.g., the lower back), have already been developed, such as instrumented versions of the commonly-used clinical test Timed Up-and-Go [46]. Using sensors embedded in the smartphones, detailed information about movement quality, as well as quantity, can be gathered. For the purpose of self-assessment, information from accelerometer and gyroscope sensors may be used to provide feedback to users about correct performance of test items. In PreventIT we aim to develop a self-assessment test battery for physical performance using smartphones worn on the body both for testing and to measure performance. For example, while performing squats, the information from the sensors may identify whether a person bends the knees and hips deeply enough. Figure 2 shows how a physical performance test application with a smartphone worn in a belt and fixed over the lumbar spine may be used to instruct and record performance of test items.

### 6.2. Delivery of Personalised Interventions

Gender, age, socio-economic class, as well as individual biological and personal factors, influence intervention preferences, uptake, and adherence [47]. Thus, the development of individually tailored interventions should be prioritised in order to increase uptake and the effects of intervention. Mobile technologies and collection of individual data on behaviour allow for the development of personally-tailored interventions with real-time feedback, and may lead to development of more versatile interventions that have the potential to increase uptake and compliance. Collection of new aspects of function and health related to performance of daily tasks during an intervention phase also opens up to better understanding of within-subject differences in uptake and may give new knowledge on variables that mediate the effects of the intervention [48].

Unobtrusive technologies, like smartphones, are well suited as a technology for delivery of interventions. The use of technology enables the development of personalised interventions with feedback to be provided in real-time to increase uptake of the intervention over time. In PreventIT, we developed an intervention with activities focusing on components of importance for active and healthy ageing, namely, balance, muscle strength, and physical activity, that are to be integrated in daily life situations and delivered using smartphones and smartwatches. The type and level of activities is individualised by algorithms developed based on existing datasets and literature, also taking into account cognitive, medical, emotional, and social factors of each individual. Motivational messages based on the health action process approach [49], using 25 different behavioural change techniques, have been developed and will be used to deliver personalised feedback in relation to each individual’s long-term goals, preferences, and activity levels.

### 6.3. Aiming at Behavioural Change

In order to promote behavioural change, people need to keep motivated over time and habits must be changed, both to remove “bad” habits and to create “good” habits. This requires the use of behavioural change theories as the basis for designing tailored motivation and feedback messages specifically relevant to older adults, and linking to behaviours monitored by the sensor system to provide behavioural feedback loops.

There is some evidence that timely text messages can support the promotion of healthy lifestyles and greater activity. King et al. [50] developed and tested three applications based on behaviour change theory that were designed to motivate adults just under 60 years of age to become less sedentary and more active. The applications used real-time feedback and focused on personalised goal-setting, behavioural feedback, social influence, including social support, modelling behaviour, competition, and positive re-enforcement with a focus on game-like feedback. All three applications received positive feedback, increased physical activity, and reduced sedentary time. What is clear is that in order to use sensors and feedback most effectively as part of a behaviour change approach, psychological models must be integrated into the development from the beginning. There are many psychological approaches, theories, and techniques that have relevance for mHealth system development, and there is a wealth of empirical evidence on which techniques work where and with whom [20,51]. In the PreventIT project we use a number of psychological theories and techniques and map the behaviour change components using a taxonomy onto the requirements of the system so as to ensure that the most effective techniques are used to devise messages delivered to individuals based on profiles and goals [52]. Thus, feedback can be personalised to fit the lifestyle, aspirations, and goals of the older person, and is based on theory that should permit delivery in a way that is optimised to bring about and maintain healthy activity.

### 6.4. Behavioural Complexity as a Measure of Risk for Age-Related Functional Decline

Preventing early signs of functional decline requires measures that are able to reveal changes in patterns of behaviour that may not be visible just by observing people’s behaviour. Healthy ageing is a complex process of adaptation to physical, social, and psychological changes. One of the most influential theories, proposed two decades ago, concerns physiological complexity and describes ageing as a process of complexity loss [53,54]. This theory postulates that healthy physiological processes are complex in that they are comprised of ongoing fluctuations with an information-rich structure. Such structural richness characterises the capacity of healthy physiological functions to detect, respond, and adapt to the innumerable perturbations and stressors in daily life. This capacity is achieved via complex interactions between multiple control systems, feedback loops, and regulatory processes that operate over multiple scales of time and space. Several studies have indicated that disease and the ageing process, itself, can be characterised by a progressive loss of complexity [55]. Loss of complexity leads to a more stereotyped behavioural pattern and at a certain level falls below a threshold such that an individual can no longer adapt to internal and external perturbations as, for example, observed with frailty.

**Figure 3:**
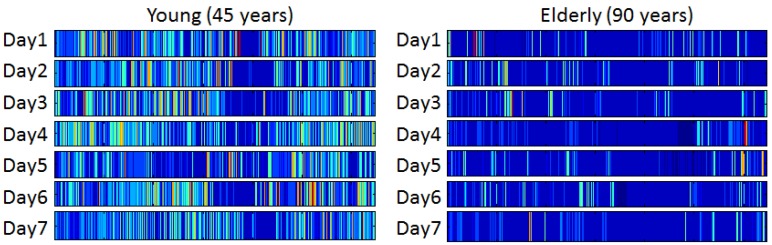
Examples of daily physical activity barcodes for a 45 years and a 90 years old participant. Dark blue colours are associated with lowest intensity activity states, and with warmer colours from green to yellow and read characterising higher intensity states. The red spectrum is associated with walking bouts at medium to high cadence.

Long-term monitoring of physiological, physical, and social dimensions of everyday life using wearable devices provide data that can be analysed and interpreted using modern concepts in physiological/behavioural research. In the PreventIT project we aim to develop a metric or a composite measure for complexity in behaviour including its temporal pattern [38] by continuous monitoring of behaviour both during the day and sleep during the night. Assessment of behavioural complexity requires monitoring over days by use of different sensors and advanced mathematical and statistical modelling with high computational power. The temporal patterns can be visualised as e.g., barcodes, where each coloured bar represents a different behavioural state at different intensity. The succession of bars with different colours and widths over a certain time period can then demonstrate the richness of the variation in behavioural patterns over time (see Figure 3). Due to its capacity to characterise the dynamics of behavioural systems, the emerging field of complex adaptive systems and its array of quantitative tools show great promise for improving our understanding of ageing, monitoring health-related behaviour, and providing outcome measures for evaluating novel interventions that promote healthy ageing.

## 7. The Way Forward

Despite being promising, mHealth technologies have not yet taken off. There are several reasons for this, including a lack of standards, making implementation in varied settings challenging [39].

Additionally, new technologies that are to be implemented in established systems and new types of health services require investment in new infrastructures. Furthermore, implementation of mHealth technologies may lead to a shift in control of the health service from the clinician to people, themselves, which implies new roles for health care personnel, and also opens up opportunities for new types and modes of delivery of interventions. Another reason may be that new technologies are constantly emerging and developing and are quickly replaced by something newer, making uptake more difficult. For the older generation, one obvious limitation is that the technologies have not been developed with this group in mind. In order to handle the change in demography and make a shift from treatment to prevention, mHealth systems need to be developed based on the needs and preferences of older adults so as to empower them to stay independent and mobile for longer.

Usable products for the end users will increase the chance for the products to be adopted and is an important issue for the development of new products. This requires that end users are involved already from the first development phase. Since most mobile health applications use existing products, such as smartphones and watches as a platform, usability is related not only to the health application, but also to the platforms on which the systems are built. Having older people in mind, the choice of functionality and usability of the platforms themselves are, therefore, of importance.

Health care intends to be evidence-based. At present, the evidence for the efficacy of mHealth services is sparse. The information gathered through mHealth systems needs to give valid, reliable and sensitive information, about risk detection, prognosis, diagnosis, and outcomes of interventions. New algorithms based on sensor measurement in mHealth systems are constantly being developed to describe new features of potential interest for assessment of health and function. To be used for health purposes, reliability and validity of such algorithms need to be carefully assessed. mHealth solutions allow for the monitoring of behaviour over time, making it possible to assess intra- and inter-individual variability in behaviour, as well as to identify change over time in response to interventions or changes in health. These new data can also help to provide insight into the efficacy of interventions, and thereby facilitate the development of more effective interventions. However, with this is new information, research is needed to better understand how to interpret the data in a health context and whether it can provide better guidance for personalised interventions.

Although the technologies used in mHealth solutions are regarded as unobtrusive and feasible for delivering personalised interventions, research is needed to assess when, where, and for whom the mHealth applications and services are efficacious [9]. In addition, we need to develop a fuller understanding of whether these technologies are indeed unobtrusive and do not, in themselves, create Hawthorne-like observer effects [56]. As most research is still at proof-of-concept or small clinical study stage, there is also a need to move to running clinical randomised controlled trials and implementing applications and services in real life. The UK-MRC Framework and subsequent Guidance provide a good development model for this [57,58].

Wearable technology with embedded sensor systems, such as smartwatches and smartphones, support development of tools for assessment of function that can be handled and organised by people themselves, instead of assessments organised and performed by health care workers. As a next step, information from self-assessment might be exchanged with health care workers and the health care system, and be used to give information about changes in function and behaviour over time and to better tailor interventions.

For many people, adoption of an active lifestyle requires a behavioural change. mHealth services may be personalised and, thus, have the potential to facilitate change in health behaviour over time. However, so far there is little knowledge about the most effective behaviour change models to be implemented using mHealth technologies, nor about long-term use of mHealth technologies and what facilitates long-term behaviour change adherence [59]. Motivation and engagement are key factors for changing behaviour and, thus, research is needed that focuses on how interventions should be designed in order to fit the users’ needs and motivations and increase uptake of interventions over time.

mHealth technologies open up opportunities for collection, calculation, and storage of large datasets about health and behaviour, including data on sensitive health conditions, location, emotion, and social interactions [60], and the possibilities of using and sharing of information are enormous. Collection and use of data for the delivery of personalised interventions and collecting and using information on a population level raises concerns about privacy, security, and confidentiality. The development of mHealth technologies and services and their possibilities of use have progressed so quickly that there is a lack of regulation on how to deal with the situation. In Europe, this will change on 25 May 2018 with the implementation of the General Data Protection Regulation (GDPR), which aims to strengthen and unify data protection for individual citizens in the European Union (EU) [61]. Although GDPR is a EU regulation, the effects will be felt far beyond the physical boundaries of the EU, since entities trading or transferring data across those boundaries will need to comply with the regulation. The main aim of GDPR is to give citizens control over their own personal data, to harmonise data protection regulations throughout European countries, and to simplify regulations for international business. This challenges research in the field of mHealth to develop methods that ensure user privacy, while at the same time also taking into account research needs [10].

The number of available mobile health applications is increasing rapidly. Important questions are then; how consumers and end users can find the apps, how they can evaluate which of them are best suited for their needs, and how they can find out whether they have undergone rigorous scientific development and testing. Initiatives on certification of health apps, such as the Xcertia guidelines [62] may be one way forward that will make product development, product performance, and security aspects more open to be evaluated and compared. Such an evaluation of current applications might also be important for people themselves, and health care providers in making decisions about willingness to pay for mobile health applications, which today remains an open question.

Current smartphones and, lately, also smartwatches, have sophisticated features and sensors that make them suitable to be used as platforms for delivering mHealthcare and services. mHealth applications are aimed at working on societal challenges related to an increasing older population and should target important aspects of active and healthy ageing. mHealth systems developed for the private market have, so far, and to a large degree, focused on cardiovascular health and have been developed with younger users in mind. In order to meet the challenges with an increasing older population and prolong the number of independent and healthy years during old age, we need mHealth systems that promote active and healthy ageing by focusing on important risk factors for age-related functional decline. mHealth solutions represent a new paradigm for delivering health services to older adults and for collecting information for the purpose of health research. Though promising, mHealth solutions are still in their infancy and there are still methodological, as well as privacy and security issues that need to be resolved. Once they are, mHealth promises opportunities for the development of new types of services and interventions that empower people to be responsible for their own health to a much larger degree, thereby changing the focus from treatment to prevention. This gives an optimistic outlook for the future.
